# 5-Chloro-2-(thio­phen-2-yl)-1-(thio­phen-2-ylmeth­yl)-1*H*-benzimidazole–6-chloro-2-(thiophen-2-yl)-1-(thiophen-2-ylmethyl)-1*H*-benzimidazole (0.94/0.06)

**DOI:** 10.1107/S1600536813024999

**Published:** 2013-09-12

**Authors:** David K. Geiger, Michael R. Nellist

**Affiliations:** a Department of Chemistry, State University of New York-College at Geneseo, 1 College Circle, Geneseo, NY 14454, USA

## Abstract

There are two independent mol­ecules in the asymmetric unit of the title compound, C_16_H_11_ClN_2_S_2_. The structure exhibits rotational disorder of the 2-thio­phen-2-yl substituent in each of the unique mol­ecules with a major:minor component ratio of 0.927 (2):0.073 (2). For one of the symmetry-unique molecules, 6.0 (2)% of the sites are occupied by the 6-chloro-isomer. The major component thio­phene rings make dihedral angles of 38.90 (12) and 36.32 (11)° with the benzimidazole rings in the two independent mol­ecules. In the crystal, mol­ecules are linked into chains parallel to [100] *via* weak C—H⋯N inter­actions.

## Related literature

For the structure of 6-chloro-2-(thio­phen-2-yl)-1-(thio­phen-2-ylmeth­yl)-1*H*-benzimidazole, see: Geiger & Nellist (2013 ▶). For the structure of the 5-bromo analogue, see: Geiger & Destefano (2012 ▶).
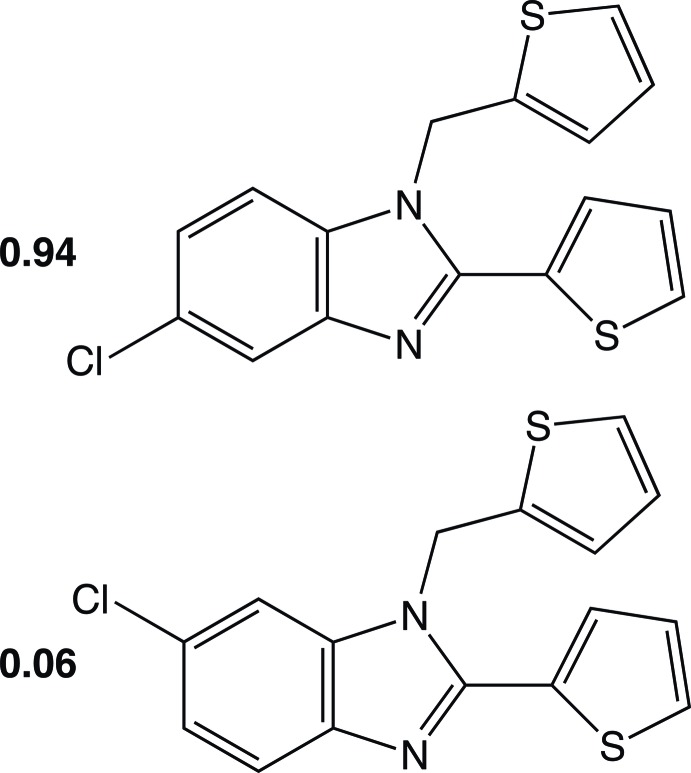


## Experimental

### 

#### Crystal data

C_16_H_11_ClN_2_S_2_
*M* *_r_* = 330.84
Monoclinic, 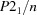

*a* = 12.7407 (11) Å
*b* = 10.5126 (8) Å
*c* = 22.955 (2) Å
β = 100.461 (3)°
*V* = 3023.4 (4) Å^3^
*Z* = 8
Mo *K*α radiation
μ = 0.52 mm^−1^
*T* = 200 K
0.80 × 0.40 × 0.20 mm

#### Data collection

Bruker SMART X2S benchtop diffractometer
Absorption correction: multi-scan (*SADABS*; Bruker, 2010 ▶) *T*_min_ = 0.62, *T*_max_ = 0.90
32145 measured reflections
5356 independent reflections
4419 reflections with *I* > 2σ(*I*)
*R*_int_ = 0.072

#### Refinement

*R*[*F* ^2^ > 2σ(*F* ^2^)] = 0.038
*wR*(*F* ^2^) = 0.093
*S* = 1.08
5356 reflections
420 parameters
227 restraints
H-atom parameters constrained
Δρ_max_ = 0.34 e Å^−3^
Δρ_min_ = −0.44 e Å^−3^

### 

Data collection: *APEX2* (Bruker, 2010 ▶); cell refinement: *SAINT* (Bruker 2010 ▶); data reduction: *SAINT*; program(s) used to solve structure: *SHELXS97* (Sheldrick, 2008 ▶); program(s) used to refine structure: *SHELXL97* (Sheldrick, 2008 ▶); molecular graphics: *PLATON* (Spek, 2009 ▶) and *Mercury* (Macrae *et al.*, 2008 ▶); software used to prepare material for publication: *publCIF* (Westrip, 2010 ▶).

## Supplementary Material

Crystal structure: contains datablock(s) global, I. DOI: 10.1107/S1600536813024999/fj2642sup1.cif

Structure factors: contains datablock(s) I. DOI: 10.1107/S1600536813024999/fj2642Isup2.hkl

Supplementary material file. DOI: 10.1107/S1600536813024999/fj2642Isup3.mol

Supplementary material file. DOI: 10.1107/S1600536813024999/fj2642Isup4.cml

Additional supplementary materials:  crystallographic information; 3D view; checkCIF report

## Figures and Tables

**Table 1:** Hydrogen-bond geometry (Å, °)

*D*—H⋯*A*	*D*—H	H⋯*A*	*D*⋯*A*	*D*—H⋯*A*
C22—H22⋯N2	0.95	2.68	3.581 (3)	159
C28—H28*B*⋯N2	0.99	2.58	3.460 (3)	148
C3^i^—H3^i^⋯N4	0.95	2.68	3.584 (3)	159
C12^i^—H12*A* ^i^⋯N4	0.99	2.62	3.514 (3)	150

Symmetry code: (i) 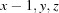.

**Fig. 1.**
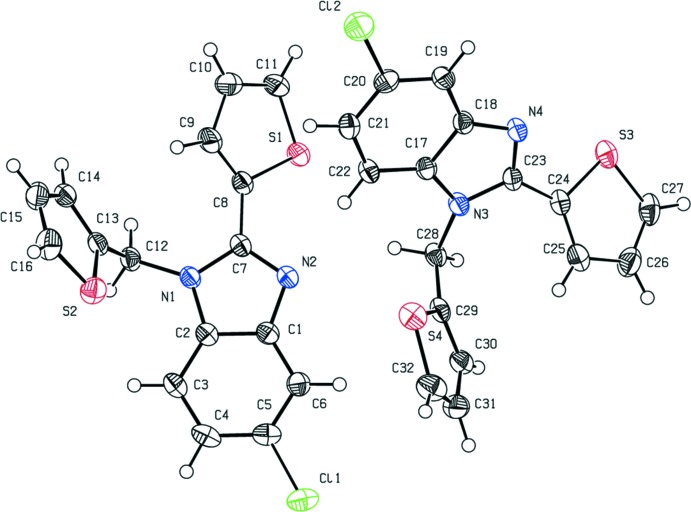
Perspective view of the title compound. Thermal parameters are drawn at the 50% probability level. Only major contributors to the disorder model are shown.

**Fig. 2.**
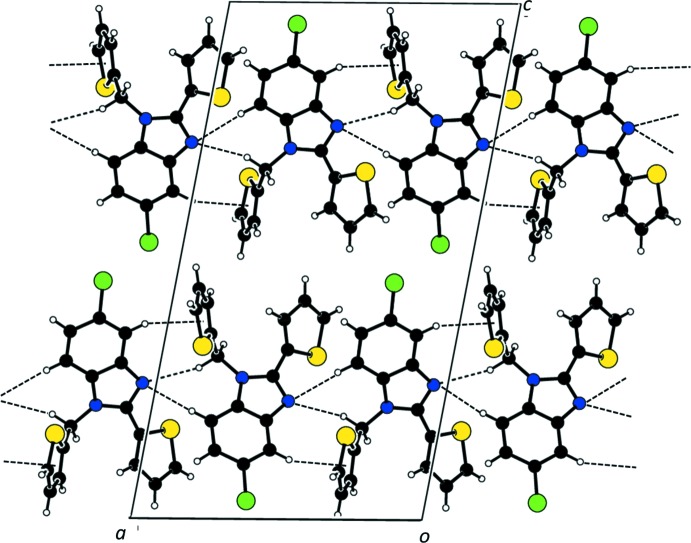
Perspective drawing showing the intermolecular contacts forming chains parallel to [1 0 0]. Only the major components of the disorder model are shown.

## supplementary crystallographic information

### 1. Comment

The title compound crystallized with two independent molecules in the asymmetric unit and is isomorphic with the corresponding 5-bromo derivative (Geiger & Destefano, 2012). Crystallization occurs with 6.0 (2)% of one of the sites (molecule 1) occupied by 6-chloro-2-(thiophen-2-yl)-1-(thiophen-2-ylmethyl)-1*H*-benzimidazole. Interestingly, the previously reported structure of the 6-chloro analogue displays co-crystallization with 3.1 (2)% of the 5-chloro derivative (Geiger & Nellist, 2013). Figure 1 shows a perspective view of the two molecules in the asymmetric unit with the atom-labeling scheme. Bond distances and angles agree well those reported for the 6-chloro analogue (Geiger & Nellist, 2013).

The benzimidazole moieties are essentially planar with r. m. s. deviation = 0.0150 Å for molecule 1 and 0.0183 Å for molecule 2. The greatest deviation from planarity is 0.0235 (19) Å for C4 in molecule 1 and 0.0271 (19) Å for C21 in molecule 2. In both molecules, the 2-thiophene substituents are rotationally disordered with a major:minor component refined-occupancy ratio of 0.927 (2):0.073 (2). The major component thiophene rings are canted 38.90 (12)° and 36.32 (11)° from the benzimidazole rings for molecules 1 and 2, respectively.

Chains of molecules parallel to [1 0 0] are held together *via* weak C—H···N and C—H···thiophene ring interactions. The motif is shown in Figure 2. The H6···thiopheneS4 centroid distance is 2.60 Å and the H19···thiopheneS2 centroid distance is 2.66 Å.

### 2. Experimental

1,2-diamine-4-chlorobenzene (6.3 mmol, 0.90 g) was dissolved in 30 mL ethanol under nitrogen. Two equivalents of 2-thiophenecarboxaldehyde (1.3 mL) was added dropwise. After three days, the solvent was removed under reduced pressure and the crude product was chromatographed (silica gel) using a mixture of 30% hexane in ethyl acetate. The first fraction produced hexagonal shaped crystals (Geiger & Nellist, 2013) and the second fraction produced needle-shaped crystals on slow evaporation. Crystals from the second fraction were used for X-ray diffraction experiments. The overall yield was 59%.

### 3. Refinement

Crystal data, data collection and structure refinement details are summarized in Table 1. All hydrogen atoms were observed in difference fourier maps. The H atoms were refined using a riding model with a C—H distance of 0.99 Å for the methylene carbon atoms and 0.95 Å for the phenyl and thiophene carbon atoms. All C—H hydrogen atom thermal parameters were set using the approximation *U*_iso_ = 1.2*U*_eq_.

The Cl and H atoms of the major and minor co-crystallization components were modeled as a disorder involving two parts, each containing a chlorine atom and a hydrogen atom. The disorder was statistically significant for only one of the molecules in the asymmetric unit. The site occupancy for the major component refined to 0.940 (2).

The 2-thiophene substituents are rotationally disordered. A model was developed in which the minor components of the thiophene rings were defined using the metrics of the major component as a guide. The disordered five-member rings were constrained to planarity using FLAT. Corresponding bond distances of the minor component and major component were set equal using SAME and corresponding thermal parameters were held the same using EADP. All atoms were refined anisotropically with hydrogen atoms in calculated positions using a riding model. With these constraints, the site occupancy of the major component refined to 0.927 (2).

### Crystal data

**Table:**

C_16_H_11_ClN_2_S_2_	*F*(000) = 1360
*M**_r_* = 330.84	*D*_x_ = 1.454 Mg m^−^^3^
Monoclinic, *P*2_1_/*n*	Mo *K*α radiation, λ = 0.71073 Å
*a* = 12.7407 (11) Å	Cell parameters from 9493 reflections
*b* = 10.5126 (8) Å	θ = 2.6–24.9°
*c* = 22.955 (2) Å	µ = 0.52 mm^−^^1^
β = 100.461 (3)°	*T* = 200 K
*V* = 3023.4 (4) Å^3^	Plate, colourless
*Z* = 8	0.80 × 0.40 × 0.20 mm

### Data collection

**Table:**

Bruker SMART X2S benchtop diffractometer	5356 independent reflections
Radiation source: XOS X-beam microfocus source	4419 reflections with *I* > 2σ(*I*)
Doubly curved silicon crystal monochromator	*R*_int_ = 0.072
ω scans	θ_max_ = 25.1°, θ_min_ = 2.1°
Absorption correction: multi-scan (*SADABS*; Bruker, 2010)	*h* = −15→14
*T*_min_ = 0.62, *T*_max_ = 0.90	*k* = −12→11
32145 measured reflections	*l* = −27→27

### Refinement

**Table:**

Refinement on *F*^2^	Primary atom site location: structure-invariant direct methods
Least-squares matrix: full	Secondary atom site location: difference Fourier map
*R*[*F*^2^ > 2σ(*F*^2^)] = 0.038	Hydrogen site location: inferred from neighbouring sites
*wR*(*F*^2^) = 0.093	H-atom parameters constrained
*S* = 1.08	*w* = 1/[σ^2^(*F*_o_^2^) + (0.0256*P*)^2^ + 1.7458*P*] where *P* = (*F*_o_^2^ + 2*F*_c_^2^)/3
5356 reflections	(Δ/σ)_max_ < 0.001
420 parameters	Δρ_max_ = 0.34 e Å^−^^3^
227 restraints	Δρ_min_ = −0.44 e Å^−^^3^

### Special details

**Table:**

Experimental. ^1^H NMR spectrum (CDCl_3_, 400 MHz, p.p.m.). 7.71 (1 H, *d*), 7.53 (1 H, *d*), 7.48 (1 H, *d*), 7.34 (1 H, *s*), 7.28 (2 H, *m*), 7.17 (1 H, *t*), 6.96 (1 H, *t*), 6.91 (1 H, *d*), 5.60 (2 H, *s*).
Geometry. All e.s.d.'s (except the e.s.d. in the dihedral angle between two l.s. planes) are estimated using the full covariance matrix. The cell e.s.d.'s are taken into account individually in the estimation of e.s.d.'s in distances, angles and torsion angles; correlations between e.s.d.'s in cell parameters are only used when they are defined by crystal symmetry. An approximate (isotropic) treatment of cell e.s.d.'s is used for estimating e.s.d.'s involving l.s. planes.
Refinement. Refinement of *F*^2^ against ALL reflections. The weighted *R*-factor *wR* and goodness of fit *S* are based on *F*^2^, conventional *R*-factors *R* are based on *F*, with *F* set to zero for negative *F*^2^. The threshold expression of *F*^2^ > σ(*F*^2^) is used only for calculating *R*-factors(gt) *etc*. and is not relevant to the choice of reflections for refinement. *R*-factors based on *F*^2^ are statistically about twice as large as those based on *F*, and *R*- factors based on ALL data will be even larger.

### Fractional atomic coordinates and isotropic or equivalent isotropic displacement parameters (Å^2^)

**Table:**

	*x*	*y*	*z*	*U*_iso_*/*U*_eq_	Occ. (<1)
Cl1	0.61379 (6)	0.64223 (7)	0.03514 (3)	0.0429 (2)	0.9401 (19)
Cl11	0.8208 (9)	0.6153 (12)	0.0775 (5)	0.049 (4)	0.0599 (19)
Cl2	0.24867 (6)	0.62779 (8)	0.45858 (3)	0.0552 (2)	
S1	0.46460 (5)	0.23990 (8)	0.31622 (3)	0.0333 (2)	0.9272 (19)
C8	0.5695 (2)	0.3464 (3)	0.32835 (12)	0.0277 (5)	0.9272 (19)
C9	0.6012 (3)	0.3670 (5)	0.3868 (2)	0.0408 (11)	0.9272 (19)
H9	0.6582	0.4227	0.4023	0.049*	0.9272 (19)
C10	0.5428 (4)	0.2989 (4)	0.42304 (16)	0.0457 (9)	0.9272 (19)
H10	0.5555	0.3036	0.4651	0.055*	0.9272 (19)
C11	0.4661 (3)	0.2258 (4)	0.39051 (14)	0.0415 (8)	0.9272 (19)
H11	0.4188	0.1728	0.4071	0.05*	0.9272 (19)
S201	0.6207 (13)	0.3828 (18)	0.3975 (8)	0.0333 (2)	0.0728 (19)
C208	0.5691 (18)	0.347 (2)	0.3258 (8)	0.0277 (5)	0.0728 (19)
C209	0.495 (3)	0.255 (4)	0.3208 (14)	0.0408 (11)	0.0728 (19)
H209	0.4615	0.2198	0.284	0.049*	0.0728 (19)
C210	0.471 (4)	0.218 (5)	0.3759 (17)	0.0457 (9)	0.0728 (19)
H210	0.4162	0.1597	0.3805	0.055*	0.0728 (19)
C211	0.537 (4)	0.274 (5)	0.4217 (14)	0.0415 (8)	0.0728 (19)
H211	0.5372	0.2563	0.4623	0.05*	0.0728 (19)
S3	−0.08034 (5)	0.24052 (8)	0.17321 (4)	0.0362 (2)	0.9272 (19)
C24	0.0189 (2)	0.3445 (3)	0.16210 (12)	0.0291 (5)	0.9272 (19)
C25	0.0157 (3)	0.3639 (5)	0.10334 (19)	0.0409 (10)	0.9272 (19)
H25	0.0635	0.419	0.0883	0.049*	0.9272 (19)
C26	−0.0651 (3)	0.2945 (3)	0.06668 (15)	0.0427 (9)	0.9272 (19)
H26	−0.0773	0.2968	0.0246	0.051*	0.9272 (19)
C27	−0.1236 (2)	0.2238 (4)	0.09864 (14)	0.0416 (8)	0.9272 (19)
H27	−0.1818	0.1713	0.0816	0.05*	0.9272 (19)
S203	0.0313 (13)	0.3664 (18)	0.0909 (8)	0.0362 (2)	0.0728 (19)
C204	0.0221 (18)	0.345 (2)	0.1633 (8)	0.0291 (5)	0.0728 (19)
C205	−0.055 (3)	0.263 (4)	0.1702 (14)	0.0409 (10)	0.0728 (19)
H205	−0.0717	0.2402	0.2076	0.049*	0.0728 (19)
C206	−0.110 (3)	0.214 (5)	0.1156 (16)	0.0427 (9)	0.0728 (19)
H206	−0.168	0.1558	0.1121	0.051*	0.0728 (19)
C207	−0.071 (4)	0.261 (5)	0.0688 (14)	0.0416 (8)	0.0728 (19)
H207	−0.0973	0.2385	0.0287	0.05*	0.0728 (19)
N1	0.70729 (13)	0.43276 (18)	0.27330 (8)	0.0266 (4)	
N2	0.53427 (13)	0.43584 (18)	0.22876 (9)	0.0289 (4)	
N3	0.19102 (13)	0.43204 (18)	0.21832 (8)	0.0274 (4)	
N4	0.04623 (13)	0.43177 (18)	0.26202 (9)	0.0284 (4)	
C1	0.59642 (16)	0.4881 (2)	0.19105 (10)	0.0268 (5)	
C2	0.70448 (16)	0.4859 (2)	0.21790 (10)	0.0260 (5)	
C3	0.78547 (17)	0.5283 (2)	0.18928 (11)	0.0318 (6)	
H3	0.8583	0.5262	0.2081	0.038*	
C4	0.75470 (18)	0.5735 (2)	0.13244 (11)	0.0345 (6)	
H4	0.8073	0.6019	0.1109	0.041*	0.9401 (19)
C5	0.64661 (19)	0.5781 (2)	0.10605 (11)	0.0315 (5)	
H5	0.628	0.6115	0.0671	0.038*	0.0599 (19)
C6	0.56597 (17)	0.5361 (2)	0.13409 (10)	0.0306 (5)	
H6	0.4932	0.5399	0.1154	0.037*	
C7	0.60260 (16)	0.4044 (2)	0.27688 (10)	0.0262 (5)	
C12	0.80522 (16)	0.4025 (2)	0.31486 (10)	0.0289 (5)	
H12A	0.8618	0.3815	0.2921	0.035*	
H12B	0.7926	0.3259	0.3377	0.035*	
C17	0.22345 (16)	0.4840 (2)	0.27401 (10)	0.0258 (5)	
C18	0.13203 (16)	0.4836 (2)	0.30049 (10)	0.0267 (5)	
C19	0.13799 (17)	0.5298 (2)	0.35781 (11)	0.0306 (5)	
H19	0.077	0.5334	0.3762	0.037*	
C20	0.23665 (18)	0.5700 (2)	0.38649 (10)	0.0321 (5)	
C21	0.32827 (17)	0.5684 (2)	0.36057 (11)	0.0317 (5)	
H21	0.3946	0.5964	0.3827	0.038*	
C22	0.32240 (16)	0.5266 (2)	0.30348 (10)	0.0292 (5)	
H22	0.3832	0.5266	0.2849	0.035*	
C23	0.08426 (15)	0.4026 (2)	0.21395 (10)	0.0262 (5)	
C28	0.26310 (16)	0.4040 (2)	0.17696 (10)	0.0284 (5)	
H28A	0.2356	0.3289	0.1529	0.034*	
H28B	0.3341	0.3815	0.2	0.034*	
S2	0.86470 (5)	0.66077 (6)	0.33429 (3)	0.03759 (17)	
C13	0.84454 (16)	0.5082 (2)	0.35767 (11)	0.0282 (5)	
C14	0.87606 (16)	0.4981 (2)	0.41784 (11)	0.0325 (6)	
H14	0.8725	0.4213	0.4392	0.039*	
C15	0.91451 (18)	0.6146 (3)	0.44476 (12)	0.0396 (6)	
H15	0.939	0.6246	0.4861	0.047*	
C16	0.91244 (19)	0.7094 (3)	0.40503 (12)	0.0418 (6)	
H16	0.935	0.7939	0.4153	0.05*	
S4	0.31336 (5)	0.66291 (6)	0.16150 (3)	0.03820 (17)	
C29	0.27602 (16)	0.5123 (2)	0.13586 (11)	0.0294 (5)	
C30	0.26814 (17)	0.5050 (2)	0.07566 (11)	0.0351 (6)	
H30	0.2493	0.4297	0.0533	0.042*	
C31	0.29151 (19)	0.6233 (3)	0.05033 (12)	0.0403 (6)	
H31	0.2902	0.6356	0.0092	0.048*	
C32	0.3156 (2)	0.7157 (3)	0.09140 (12)	0.0431 (7)	
H32	0.332	0.8008	0.0823	0.052*	

### Atomic displacement parameters (Å^2^)

**Table:**

	*U*^11^	*U*^22^	*U*^33^	*U*^12^	*U*^13^	*U*^23^
Cl1	0.0563 (4)	0.0435 (4)	0.0311 (4)	0.0043 (3)	0.0138 (3)	0.0081 (3)
Cl11	0.051 (6)	0.063 (9)	0.034 (7)	−0.011 (5)	0.009 (5)	0.000 (6)
Cl2	0.0515 (4)	0.0819 (6)	0.0331 (4)	−0.0096 (4)	0.0102 (3)	−0.0183 (4)
S1	0.0295 (4)	0.0352 (4)	0.0347 (4)	−0.0119 (3)	0.0046 (3)	0.0021 (3)
C8	0.0231 (10)	0.0265 (13)	0.0337 (14)	−0.0024 (9)	0.0057 (9)	0.0012 (11)
C9	0.033 (2)	0.052 (2)	0.038 (2)	−0.0188 (16)	0.0054 (16)	−0.0017 (18)
C10	0.0437 (16)	0.063 (3)	0.0309 (16)	−0.0155 (16)	0.0080 (12)	0.0035 (15)
C11	0.0361 (14)	0.0511 (19)	0.038 (2)	−0.0146 (13)	0.0099 (15)	0.0091 (17)
S201	0.0295 (4)	0.0352 (4)	0.0347 (4)	−0.0119 (3)	0.0046 (3)	0.0021 (3)
C208	0.0231 (10)	0.0265 (13)	0.0337 (14)	−0.0024 (9)	0.0057 (9)	0.0012 (11)
C209	0.033 (2)	0.052 (2)	0.038 (2)	−0.0188 (16)	0.0054 (16)	−0.0017 (18)
C210	0.0437 (16)	0.063 (3)	0.0309 (16)	−0.0155 (16)	0.0080 (12)	0.0035 (15)
C211	0.0361 (14)	0.0511 (19)	0.038 (2)	−0.0146 (13)	0.0099 (15)	0.0091 (17)
S3	0.0271 (4)	0.0369 (5)	0.0435 (4)	−0.0089 (3)	0.0037 (3)	−0.0040 (3)
C24	0.0210 (10)	0.0310 (13)	0.0346 (14)	0.0000 (9)	0.0032 (9)	−0.0021 (11)
C25	0.0257 (19)	0.054 (2)	0.043 (3)	−0.0045 (15)	0.0062 (13)	−0.0012 (19)
C26	0.0367 (14)	0.053 (3)	0.0351 (16)	0.0008 (15)	−0.0030 (12)	−0.0060 (15)
C27	0.0280 (14)	0.0468 (19)	0.046 (2)	−0.0060 (13)	−0.0046 (13)	−0.0113 (17)
S203	0.0271 (4)	0.0369 (5)	0.0435 (4)	−0.0089 (3)	0.0037 (3)	−0.0040 (3)
C204	0.0210 (10)	0.0310 (13)	0.0346 (14)	0.0000 (9)	0.0032 (9)	−0.0021 (11)
C205	0.0257 (19)	0.054 (2)	0.043 (3)	−0.0045 (15)	0.0062 (13)	−0.0012 (19)
C206	0.0367 (14)	0.053 (3)	0.0351 (16)	0.0008 (15)	−0.0030 (12)	−0.0060 (15)
C207	0.0280 (14)	0.0468 (19)	0.046 (2)	−0.0060 (13)	−0.0046 (13)	−0.0113 (17)
N1	0.0219 (8)	0.0274 (11)	0.0308 (11)	−0.0016 (7)	0.0059 (8)	0.0029 (9)
N2	0.0232 (9)	0.0330 (11)	0.0311 (11)	−0.0019 (8)	0.0067 (8)	0.0024 (9)
N3	0.0216 (9)	0.0320 (11)	0.0291 (11)	−0.0019 (8)	0.0059 (7)	−0.0055 (9)
N4	0.0207 (9)	0.0320 (11)	0.0325 (11)	−0.0012 (8)	0.0048 (8)	−0.0009 (9)
C1	0.0269 (11)	0.0248 (13)	0.0299 (13)	0.0019 (9)	0.0086 (9)	−0.0013 (10)
C2	0.0261 (10)	0.0214 (12)	0.0315 (13)	0.0016 (9)	0.0078 (9)	−0.0009 (10)
C3	0.0259 (11)	0.0303 (14)	0.0413 (15)	−0.0001 (9)	0.0118 (10)	0.0007 (11)
C4	0.0391 (13)	0.0263 (14)	0.0438 (16)	−0.0008 (10)	0.0230 (11)	0.0012 (12)
C5	0.0433 (13)	0.0223 (13)	0.0317 (14)	0.0043 (10)	0.0145 (11)	0.0018 (11)
C6	0.0294 (11)	0.0306 (14)	0.0324 (14)	0.0053 (10)	0.0071 (10)	0.0012 (11)
C7	0.0230 (10)	0.0262 (13)	0.0299 (13)	−0.0024 (9)	0.0065 (9)	−0.0026 (10)
C12	0.0217 (10)	0.0290 (13)	0.0356 (14)	0.0020 (9)	0.0042 (9)	0.0053 (11)
C17	0.0239 (10)	0.0252 (13)	0.0283 (13)	0.0015 (9)	0.0047 (9)	−0.0006 (10)
C18	0.0224 (10)	0.0266 (13)	0.0307 (13)	0.0017 (9)	0.0038 (9)	0.0019 (10)
C19	0.0262 (11)	0.0350 (14)	0.0320 (14)	0.0040 (10)	0.0093 (10)	−0.0001 (11)
C20	0.0376 (12)	0.0325 (14)	0.0259 (13)	0.0004 (10)	0.0049 (10)	−0.0035 (11)
C21	0.0269 (11)	0.0323 (14)	0.0346 (14)	−0.0033 (10)	0.0020 (10)	−0.0024 (11)
C22	0.0213 (10)	0.0323 (14)	0.0346 (14)	0.0005 (9)	0.0070 (9)	−0.0021 (11)
C23	0.0202 (10)	0.0260 (13)	0.0315 (13)	−0.0002 (9)	0.0022 (9)	0.0013 (10)
C28	0.0233 (10)	0.0315 (13)	0.0314 (13)	0.0000 (9)	0.0078 (9)	−0.0082 (11)
S2	0.0398 (3)	0.0302 (4)	0.0410 (4)	−0.0011 (3)	0.0027 (3)	0.0069 (3)
C13	0.0192 (10)	0.0301 (13)	0.0359 (14)	0.0002 (9)	0.0067 (9)	0.0052 (11)
C14	0.0259 (11)	0.0348 (14)	0.0367 (15)	−0.0021 (10)	0.0056 (10)	0.0079 (12)
C15	0.0312 (12)	0.0489 (17)	0.0368 (15)	0.0022 (11)	0.0013 (10)	−0.0028 (13)
C16	0.0397 (13)	0.0332 (15)	0.0501 (17)	−0.0011 (11)	0.0018 (12)	−0.0039 (13)
S4	0.0412 (3)	0.0324 (4)	0.0424 (4)	0.0001 (3)	0.0117 (3)	−0.0055 (3)
C29	0.0193 (10)	0.0344 (14)	0.0353 (14)	0.0007 (9)	0.0070 (9)	−0.0054 (11)
C30	0.0282 (11)	0.0452 (16)	0.0336 (14)	−0.0016 (11)	0.0100 (10)	−0.0029 (12)
C31	0.0371 (13)	0.0501 (18)	0.0352 (15)	0.0061 (12)	0.0103 (11)	0.0070 (13)
C32	0.0464 (14)	0.0340 (15)	0.0520 (18)	0.0050 (12)	0.0176 (13)	0.0077 (14)

### Geometric parameters (Å, º)

**Table:**

Cl1—C5	1.741 (2)	N3—C17	1.383 (3)
Cl11—C4	1.696 (10)	N3—C28	1.466 (3)
Cl2—C20	1.743 (2)	N4—C23	1.319 (3)
S1—C11	1.708 (3)	N4—C18	1.386 (3)
S1—C8	1.728 (3)	C1—C6	1.390 (3)
C8—C9	1.347 (5)	C1—C2	1.403 (3)
C8—C7	1.457 (3)	C2—C3	1.393 (3)
C9—C10	1.408 (5)	C3—C4	1.377 (3)
C9—H9	0.95	C3—H3	0.95
C10—C11	1.355 (4)	C4—C5	1.401 (3)
C10—H10	0.95	C4—H4	0.95
C11—H11	0.95	C5—C6	1.380 (3)
S201—C208	1.701 (16)	C5—H5	0.95
S201—C211	1.719 (16)	C6—H6	0.95
C208—C209	1.337 (16)	C12—C13	1.507 (3)
C208—C7	1.410 (17)	C12—H12A	0.99
C209—C210	1.410 (16)	C12—H12B	0.99
C209—H209	0.95	C17—C22	1.392 (3)
C210—C211	1.359 (16)	C17—C18	1.408 (3)
C210—H210	0.95	C18—C19	1.392 (3)
C211—H211	0.95	C19—C20	1.375 (3)
S3—C27	1.709 (3)	C19—H19	0.95
S3—C24	1.725 (3)	C20—C21	1.403 (3)
C24—C25	1.358 (5)	C21—C22	1.371 (3)
C24—C23	1.457 (3)	C21—H21	0.95
C25—C26	1.410 (5)	C22—H22	0.95
C25—H25	0.95	C28—C29	1.507 (3)
C26—C27	1.358 (4)	C28—H28A	0.99
C26—H26	0.95	C28—H28B	0.99
C27—H27	0.95	S2—C16	1.706 (3)
S203—C204	1.703 (16)	S2—C13	1.725 (2)
S203—C207	1.717 (16)	C13—C14	1.371 (3)
C204—C205	1.340 (16)	C14—C15	1.418 (4)
C204—C23	1.419 (16)	C14—H14	0.95
C205—C206	1.415 (16)	C15—C16	1.348 (4)
C205—H205	0.95	C15—H15	0.95
C206—C207	1.357 (15)	C16—H16	0.95
C206—H206	0.95	S4—C32	1.707 (3)
C207—H207	0.95	S4—C29	1.726 (2)
N1—C2	1.383 (3)	C29—C30	1.369 (3)
N1—C7	1.384 (3)	C30—C31	1.427 (4)
N1—C12	1.461 (3)	C30—H30	0.95
N2—C7	1.319 (3)	C31—C32	1.350 (4)
N2—C1	1.388 (3)	C31—H31	0.95
N3—C23	1.381 (3)	C32—H32	0.95
			
C11—S1—C8	91.57 (14)	C6—C5—Cl1	119.14 (19)
C9—C8—C7	131.5 (3)	C4—C5—Cl1	117.90 (18)
C9—C8—S1	110.3 (2)	C6—C5—H5	118.5
C7—C8—S1	118.0 (2)	C4—C5—H5	118.5
C8—C9—C10	114.3 (3)	Cl1—C5—H5	1.2
C8—C9—H9	122.8	C5—C6—C1	116.8 (2)
C10—C9—H9	122.8	C5—C6—H6	121.6
C11—C10—C9	111.6 (3)	C1—C6—H6	121.6
C11—C10—H10	124.2	N2—C7—N1	113.1 (2)
C9—C10—H10	124.2	N2—C7—C208	121.8 (10)
C10—C11—S1	112.2 (2)	N1—C7—C208	125.1 (10)
C10—C11—H11	123.9	N2—C7—C8	122.7 (2)
S1—C11—H11	123.9	N1—C7—C8	124.2 (2)
C208—S201—C211	90.7 (11)	C208—C7—C8	1.3 (12)
C209—C208—C7	123 (2)	N1—C12—C13	114.12 (18)
C209—C208—S201	112.8 (13)	N1—C12—H12A	108.7
C7—C208—S201	123.7 (19)	C13—C12—H12A	108.7
C208—C209—C210	112.7 (16)	N1—C12—H12B	108.7
C208—C209—H209	123.6	C13—C12—H12B	108.7
C210—C209—H209	123.6	H12A—C12—H12B	107.6
C211—C210—C209	111.7 (17)	N3—C17—C22	132.0 (2)
C211—C210—H210	124.2	N3—C17—C18	105.43 (18)
C209—C210—H210	124.2	C22—C17—C18	122.6 (2)
C210—C211—S201	111.9 (15)	N4—C18—C19	129.87 (19)
C210—C211—H211	124.1	N4—C18—C17	110.2 (2)
S201—C211—H211	124.1	C19—C18—C17	120.0 (2)
C27—S3—C24	91.65 (14)	C20—C19—C18	116.6 (2)
C25—C24—C23	131.2 (3)	C20—C19—H19	121.7
C25—C24—S3	110.6 (2)	C18—C19—H19	121.7
C23—C24—S3	118.1 (2)	C19—C20—C21	123.5 (2)
C24—C25—C26	113.7 (3)	C19—C20—Cl2	118.46 (18)
C24—C25—H25	123.1	C21—C20—Cl2	118.05 (18)
C26—C25—H25	123.1	C22—C21—C20	120.3 (2)
C27—C26—C25	111.9 (3)	C22—C21—H21	119.9
C27—C26—H26	124.0	C20—C21—H21	119.9
C25—C26—H26	124.0	C21—C22—C17	117.1 (2)
C26—C27—S3	112.1 (2)	C21—C22—H22	121.5
C26—C27—H27	123.9	C17—C22—H22	121.5
S3—C27—H27	123.9	N4—C23—N3	113.17 (19)
C204—S203—C207	91.1 (11)	N4—C23—C204	123.4 (10)
C205—C204—C23	120 (2)	N3—C23—C204	123.5 (10)
C205—C204—S203	112.5 (13)	N4—C23—C24	122.6 (2)
C23—C204—S203	128.0 (19)	N3—C23—C24	124.3 (2)
C204—C205—C206	112.7 (16)	C204—C23—C24	1.0 (9)
C204—C205—H205	123.7	N3—C28—C29	113.96 (18)
C206—C205—H205	123.7	N3—C28—H28A	108.8
C207—C206—C205	112.1 (17)	C29—C28—H28A	108.8
C207—C206—H206	124.0	N3—C28—H28B	108.8
C205—C206—H206	124.0	C29—C28—H28B	108.8
C206—C207—S203	111.6 (15)	H28A—C28—H28B	107.7
C206—C207—H207	124.2	C16—S2—C13	91.71 (13)
S203—C207—H207	124.2	C14—C13—C12	127.0 (2)
C2—N1—C7	106.10 (18)	C14—C13—S2	110.64 (18)
C2—N1—C12	124.36 (17)	C12—C13—S2	122.21 (18)
C7—N1—C12	129.22 (19)	C13—C14—C15	112.8 (2)
C7—N2—C1	104.91 (17)	C13—C14—H14	123.6
C23—N3—C17	106.32 (17)	C15—C14—H14	123.6
C23—N3—C28	129.24 (19)	C16—C15—C14	112.4 (2)
C17—N3—C28	124.14 (17)	C16—C15—H15	123.8
C23—N4—C18	104.92 (17)	C14—C15—H15	123.8
N2—C1—C6	129.6 (2)	C15—C16—S2	112.5 (2)
N2—C1—C2	110.2 (2)	C15—C16—H16	123.8
C6—C1—C2	120.2 (2)	S2—C16—H16	123.8
N1—C2—C3	131.7 (2)	C32—S4—C29	91.59 (13)
N1—C2—C1	105.68 (18)	C30—C29—C28	126.4 (2)
C3—C2—C1	122.6 (2)	C30—C29—S4	111.14 (19)
C4—C3—C2	116.7 (2)	C28—C29—S4	122.31 (17)
C4—C3—H3	121.6	C29—C30—C31	112.3 (2)
C2—C3—H3	121.6	C29—C30—H30	123.8
C3—C4—C5	120.7 (2)	C31—C30—H30	123.8
C3—C4—Cl11	134.4 (4)	C32—C31—C30	112.4 (2)
C5—C4—Cl11	104.5 (4)	C32—C31—H31	123.8
C3—C4—H4	119.7	C30—C31—H31	123.8
C5—C4—H4	119.7	C31—C32—S4	112.6 (2)
Cl11—C4—H4	16.2	C31—C32—H32	123.7
C6—C5—C4	123.0 (2)	S4—C32—H32	123.7
			
C11—S1—C8—C9	0.0 (3)	C9—C8—C7—N1	41.4 (5)
C11—S1—C8—C7	−175.5 (2)	S1—C8—C7—N1	−144.2 (2)
C7—C8—C9—C10	174.6 (3)	C9—C8—C7—C208	180.100
S1—C8—C9—C10	−0.1 (5)	S1—C8—C7—C208	−10.40
C8—C9—C10—C11	0.2 (6)	C2—N1—C12—C13	91.6 (3)
C9—C10—C11—S1	−0.2 (5)	C7—N1—C12—C13	−95.8 (3)
C8—S1—C11—C10	0.1 (3)	C23—N3—C17—C22	177.4 (2)
C211—S201—C208—C209	−1 (3)	C28—N3—C17—C22	3.2 (4)
C211—S201—C208—C7	179.9 (16)	C23—N3—C17—C18	−0.4 (2)
C7—C208—C209—C210	−177 (2)	C28—N3—C17—C18	−174.5 (2)
S201—C208—C209—C210	4 (4)	C23—N4—C18—C19	−179.4 (2)
C208—C209—C210—C211	−6 (5)	C23—N4—C18—C17	−0.3 (3)
C209—C210—C211—S201	5 (5)	N3—C17—C18—N4	0.4 (3)
C208—S201—C211—C210	−2 (4)	C22—C17—C18—N4	−177.6 (2)
C27—S3—C24—C25	0.5 (3)	N3—C17—C18—C19	179.6 (2)
C27—S3—C24—C23	176.1 (2)	C22—C17—C18—C19	1.6 (4)
C23—C24—C25—C26	−175.6 (3)	N4—C18—C19—C20	176.7 (2)
S3—C24—C25—C26	−0.8 (5)	C17—C18—C19—C20	−2.3 (3)
C24—C25—C26—C27	0.7 (5)	C18—C19—C20—C21	1.2 (4)
C25—C26—C27—S3	−0.4 (4)	C18—C19—C20—Cl2	−179.58 (17)
C24—S3—C27—C26	−0.1 (3)	C19—C20—C21—C22	0.7 (4)
C207—S203—C204—C205	−1 (3)	Cl2—C20—C21—C22	−178.49 (19)
C207—S203—C204—C23	−179.9 (18)	C20—C21—C22—C17	−1.5 (3)
C23—C204—C205—C206	180 (2)	N3—C17—C22—C21	−177.1 (2)
S203—C204—C205—C206	0 (3)	C18—C17—C22—C21	0.3 (3)
C204—C205—C206—C207	0 (5)	C18—N4—C23—N3	0.1 (3)
C205—C206—C207—S203	−1 (5)	C18—N4—C23—C204	179.0 (12)
C204—S203—C207—C206	1 (4)	C18—N4—C23—C24	179.8 (2)
C7—N2—C1—C6	179.3 (2)	C17—N3—C23—N4	0.2 (3)
C7—N2—C1—C2	0.6 (2)	C28—N3—C23—N4	174.0 (2)
C7—N1—C2—C3	−177.6 (2)	C17—N3—C23—C204	−178.8 (12)
C12—N1—C2—C3	−3.6 (4)	C28—N3—C23—C204	−5.0 (12)
C7—N1—C2—C1	0.8 (2)	C17—N3—C23—C24	−179.5 (2)
C12—N1—C2—C1	174.8 (2)	C28—N3—C23—C24	−5.7 (4)
N2—C1—C2—N1	−0.9 (2)	C205—C204—C23—N4	−33 (2)
C6—C1—C2—N1	−179.8 (2)	S203—C204—C23—N4	146.3 (13)
N2—C1—C2—C3	177.7 (2)	C205—C204—C23—N3	146 (2)
C6—C1—C2—C3	−1.2 (3)	S203—C204—C23—N3	−35 (2)
N1—C2—C3—C4	178.1 (2)	C205—C204—C23—C24	−70.80
C1—C2—C3—C4	0.0 (3)	S203—C204—C23—C24	110.80
C2—C3—C4—C5	1.3 (3)	C25—C24—C23—N4	141.0 (4)
C2—C3—C4—Cl11	−170.1 (6)	S3—C24—C23—N4	−33.6 (3)
C3—C4—C5—C6	−1.4 (4)	C25—C24—C23—N3	−39.4 (5)
Cl11—C4—C5—C6	172.3 (5)	S3—C24—C23—N3	146.1 (2)
C3—C4—C5—Cl1	177.54 (19)	C25—C24—C23—C204	−80.80
Cl11—C4—C5—Cl1	−8.8 (5)	S3—C24—C23—C204	110.80
C4—C5—C6—C1	0.1 (3)	C23—N3—C28—C29	96.9 (3)
Cl1—C5—C6—C1	−178.77 (17)	C17—N3—C28—C29	−90.3 (3)
N2—C1—C6—C5	−177.5 (2)	N1—C12—C13—C14	132.0 (2)
C2—C1—C6—C5	1.1 (3)	N1—C12—C13—S2	−52.9 (2)
C1—N2—C7—N1	−0.1 (3)	C16—S2—C13—C14	−1.03 (17)
C1—N2—C7—C208	−179.4 (12)	C16—S2—C13—C12	−176.80 (18)
C1—N2—C7—C8	179.5 (2)	C12—C13—C14—C15	176.60 (19)
C2—N1—C7—N2	−0.5 (3)	S2—C13—C14—C15	1.1 (2)
C12—N1—C7—N2	−174.1 (2)	C13—C14—C15—C16	−0.6 (3)
C2—N1—C7—C208	178.9 (13)	C14—C15—C16—S2	−0.2 (3)
C12—N1—C7—C208	5.3 (13)	C13—S2—C16—C15	0.73 (19)
C2—N1—C7—C8	179.9 (2)	N3—C28—C29—C30	−131.2 (2)
C12—N1—C7—C8	6.4 (4)	N3—C28—C29—S4	53.8 (2)
C209—C208—C7—N2	41 (2)	C32—S4—C29—C30	1.10 (18)
S201—C208—C7—N2	−140.1 (12)	C32—S4—C29—C28	176.82 (18)
C209—C208—C7—N1	−138 (2)	C28—C29—C30—C31	−176.18 (19)
S201—C208—C7—N1	41 (2)	S4—C29—C30—C31	−0.7 (2)
C209—C208—C7—C8	180.100	C29—C30—C31—C32	−0.3 (3)
S201—C208—C7—C8	0.40	C30—C31—C32—S4	1.1 (3)
C9—C8—C7—N2	−138.1 (4)	C29—S4—C32—C31	−1.3 (2)
S1—C8—C7—N2	36.2 (3)		

### Hydrogen-bond geometry (Å, º)

**Table:**

*D*—H···*A*	*D*—H	H···*A*	*D*···*A*	*D*—H···*A*
C22—H22···N2	0.95	2.68	3.581 (3)	159
C28—H28*B*···N2	0.99	2.58	3.460 (3)	148
C3^i^—H3^i^···N4	0.95	2.68	3.584 (3)	159
C12^i^—H12*A*^i^···N4	0.99	2.62	3.514 (3)	150

Symmetry code: (i) *x*−1, *y*, *z*.
